# Afghan women and children’s health: Three main challenges under Taliban and COVID-19

**DOI:** 10.7189/jogh.11.03126

**Published:** 2021-12-18

**Authors:** Cecilia Acuti Martellucci, Shohra Qaderi, Tetsuya Tanimoto, Akihiko Ozaki

**Affiliations:** 1 Department of Medical Sciences, University of Ferrara, Ferrara, Italy; 2 Student Research Committee, School of Medicine, Shahid Beheshti University of Medical Sciences, Tehran, Iran; 3 Medical Governance Research Institute, Tokyo, Japan; 4 Department of Breast Surgery, Jyoban Hospital of Tokiwa Foundation, Fukushima, Japan

In August 2021 the Taliban took over Afghanistan, as the country was battling the third wave of COVID-19, with 155 132 confirmed cases and 7128 deaths (September 25, 2021) [1]. Despite significant progress in the health care system over the last 17 years under the Islamic Republic of Afghanistan, largely attributable to the cooperation with international organizations, Afghanistan still has one of the weakest health systems in the world. Notably, the COVID-19 pandemic has exacerbated poverty and inequality, and the Taliban have added massive pressure to already overwhelmed social and health systems, resulting in a humanitarian and health crisis [2].

On August 24, the World Bank has suspended payments to Afghanistan, and it is feared that the most serious consequences of the lack of funds will primarily and most rapidly affect the well-being of women and children. Indeed, if economic hardship adds to the present crisis, and if adequate humanitarian assistance is not rapidly organized, Afghanistan risks reversing the achievements in child and maternal health obtained over the last decades [3]. The major source of concern is that the Taliban adhere to a fundamentalist interpretation of the Islamic inheritance (Islam Sharia), which severely restricts women’s freedom of movement, access to health care, education, and employment. In this context, the most compelling challenges for protecting the well-being of women and children are arguably 3-fold.

## ACCESS TO MEDICAL CARE

The first one is represented by the likely decline in access to medical care, driven mainly by two factors. On one hand, Afghanistan depends heavily on imports, including medical equipment and supplies. [4]. These have been interrupted, due to the border closures and the upheaval at Kabul airport, and as a result, as one example, children with hemophilia and thalassemia are in urgent need for life-saving imported coagulation factors and transfusions [4]. On the other hand, the double threat of the Taliban and the pandemic is a pressing concern for women, whose health outcomes were seen to deteriorate more than men both during Taliban rule and the COVID-19 pandemic, mainly because of upstream social determinants of health such as systemic inequities in the access to education, work, and therefore the opportunity to earn an independent livelihood · [3]. Indeed, while both male and female patients can seek health services, females are particularly fearful of leaving their homes, except for life-threatening conditions. This practice, spurred mainly by the religious heritage, is inevitably magnified by the reinstatement of Sharia law, whose byproducts include an appeal for separate hospital treatment for men and women. Clearly, the implementation of similar measures would increase the risk of home delivery with no prenatal or natal care, resulting in higher maternal and neonatal mortality rates, the latter being driven, among other conditions, by a dramatic increase in neonatal Tetanus [5].

**Figure:**
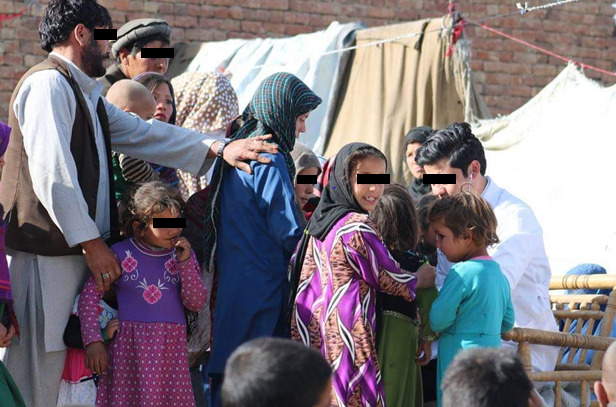
Photo: Doctor providing care to internally displaced people in a camp (from Shohra Qaderi’s own collection, used with permission).

## INTERNALLY DISPLACED PERSONS

The second challenge is managing the displacement of Afghan people, which is likely to exacerbate both conflict and disease outbreaks. Externally displaced refugees are rapidly increasing: while over 100 000 Afghans have been evacuated by plane in the two weeks after the Taliban seized Kabul, many more are currently seeking to leave the country, also through land borders [6]. Both neighboring and further countries, however, are accepting limited numbers of asylum seekers, often with the condition of repatriation [6]. Further, the victims of the August 26 terrorist attacks at Kabul airport are a clear reminder of these groups’ vulnerability amidst crisis, and of the urgent need to reinstate security. However, the main emergency is represented by internally displaced people, which increased by 558 123 individuals just in 2021, reaching a total of almost four million [7]. Such numbers can rapidly exacerbate the existing shortages of adequate shelter, food supplies, personal protective equipment, and treating capacity of the host provinces, and also insecurity, especially for the poor [7].

Again, access to health care is one of the main issues for displaced people, and women and children usually suffer the most serious consequences, even more so since women generally follow the Afghan culture of not seeking health services from a male practitioner, further delaying diagnoses and life-saving treatment.

In fact, internally displaced women and children are especially threatened in the areas of violence, mental health, reproductive health (such as pregnancy due to sexual violence), nutrition, sanitation, and infectious diseases [8]. COVID-19, in particular, is spreading quickly amongst refugees and large outbreaks of the disease are expected to occur. Similar to women, the permanence of children in displacement camps has been associated with high rates of diarrhea, scabies, and lice, as well as malnutrition, domestic violence, and psychological trauma [9,10]. In addition, mortality rates were found to be higher among displaced women than in refugees, and a tangible fear is that the lack of reproductive health services, including family planning and treatment of sexually transmitted diseases, may cause the largest part of morbidity and mortality in these women [11,12].

## IMMUNIZATION

Finally, the third challenge is immunization, the campaigns for which are currently on the brink of complete cessation [13]. The resurgence of vaccine-preventable diseases is likely to become possible, and the situation is especially precarious because Afghanistan and Pakistan are well known as the only remaining polio-endemic countries. While immunization efforts have already been hit badly by the COVID-19 pandemic, many routine appointments including childhood and adulthood vaccinations, including poliomyelitis and COVID-19 itself, were halted due to violence soars and staff cuts to these services. As poliovirus circulation remains high in the inaccessible southern and eastern regions, the current insecurity threatens to spread the infection to provinces that have remained polio-free for a long time, complicating the achievement of global polio eradication.

## CONCLUSIONS

When considering the Taliban resurgence, we should be mindful that the three listed challenges all entail clear immediate threats to health, but also equally clear long-term increases in morbidity and mortality driven by (1) untreated chronic diseases in the case of the insufficient access to health care, (2) reproductive and mental health issues and malnutrition in the case of the traumatic living conditions of internally displaced people, and (3) recurrent outbreaks of vaccine-preventable diseases and important activity backlogs in the case of the faltering immunization coverages. Such extreme consequences must be avoided, therefore every effort should be made by international actors to reinstate and maintain the sustainable provision of humanitarian and health care aid to Afghan people within this exceptional crisis, prioritizing the security, and physical and mental health of women and children. This will require not only distributing accountable aid funding, but also mediating with the Taliban for the respect of the human rights of women and children.
